# Noncirrhotic Portal Hypertension and Diagnostic Pitfalls in Severe Polycystic Liver Disease: A Case Report

**DOI:** 10.7759/cureus.117873

**Published:** 2026-10-13

**Authors:** Sahana Natesan, M. Kulebra, Stephanie Midgley

**Affiliations:** 1 Emergency Medicine, Touro College of Osteopathic Medicine, Middletown, USA; 2 Emergency Medicine, Vassar Brothers Medical Center, Poughkeepsie, USA

**Keywords:** ascites, noncirrhotic portal hypertension, point-of-care-ultrasound, polycystic kidney and liver disease, spontaneous bacterial peritonitis (sbp)

## Abstract

Polycystic liver disease (PLD) is a rare inherited disorder characterized by progressive hepatic cyst formation and, in severe cases, noncirrhotic portal hypertension. Although ascites is a recognized complication of advanced PLD, diagnostic frameworks for ascites and spontaneous bacterial peritonitis (SBP) were developed primarily in patients with cirrhosis and may not be directly applicable to noncirrhotic disease. We present the case of a 60-year-old male with severe PLD who developed recurrent massive ascites following hepatic cyst surgery. Imaging demonstrated extensive hepatic cystic disease with portal vein compression and hepatic venous outflow obstruction. Ascitic fluid demonstrated a high serum-ascites albumin gradient and elevated protein, consistent with noncirrhotic portal hypertension. Despite a polymorphonuclear leukocyte (PMN) count of only 16 cells/mm³, broth-only growth of three oropharyngeal commensal organisms prompted treatment for presumed SBP; subsequent negative cultures and the absence of neutrocytic ascites favored contamination or transient bacterascites. Point-of-care ultrasound (POCUS) facilitated rapid identification of free fluid despite extensive cystic hepatic anatomy and enabled safe paracentesis by identifying appropriate fluid pockets. This case highlights the importance of contextualizing cirrhosis-derived diagnostic frameworks when evaluating noncirrhotic ascites and demonstrates the value of POCUS as a practical diagnostic and procedural tool in patients with severe PLD.

## Introduction

Polycystic liver disease (PLD) is a group of inherited disorders characterized by progressive development of fluid-filled hepatic cysts arising from biliary epithelium [1]. PLD occurs either as isolated autosomal dominant polycystic liver disease (ADPLD), with an estimated prevalence of 1-10 per million, or in association with autosomal dominant polycystic kidney disease (ADPKD), in which hepatic cysts are present in over 80% of patients older than 40 years [1-3]. PLD demonstrates a strong female predominance, with estrogen exposure identified as a major contributor to cyst growth and disease severity [2-4]. Severe symptomatic PLD in males without concurrent polycystic kidney disease is uncommon and underrepresented in the literature [5,6].

Although most patients remain asymptomatic despite substantial cyst burden, a subset develop complications from mass effect, including portal hypertension and ascites [1,7]. In the absence of other chronic liver disease, this ascites is noncirrhotic. Diagnostic frameworks for ascites, however, were developed and validated almost exclusively in cirrhotic populations [8,9]. For example, spontaneous bacterial peritonitis (SBP) is defined by an ascitic fluid polymorphonuclear leukocyte (PMN) count ≥250 cells/mm³, a threshold derived from patients with cirrhosis [8,9]. Whether such criteria apply to noncirrhotic ascites, particularly in PLD, remains uncertain.

Management of symptomatic PLD also presents challenges. Surgical fenestration and cyst resection may provide symptomatic relief but are associated with substantial recurrence and postoperative complication rates, including ascites, bile leak, and pleural effusion [1,10]. Postoperative ascites has been reported in up to 40% of fenestration cases and is more common in diffuse PLD compared with solitary hepatic cysts [10,11]. In patients with massive cystic hepatomegaly, even routine bedside procedures, such as paracentesis, can be technically challenging. 

We present a case of a 60-year-old male with severe PLD who developed massive noncirrhotic ascites following prior hepatic cyst surgery. During evaluation, ascitic fluid culture results were misinterpreted as SBP, resulting in hospitalization and prolonged antibiotic treatment. The case highlights the mechanisms of noncirrhotic portal hypertension in PLD, the diagnostic pitfalls of applying cirrhosis-based ascitic fluid interpretation frameworks to noncirrhotic disease, and the utility of point-of-care ultrasound (POCUS) in both diagnosis and procedural guidance. 

## Case presentation

A 60-year-old male with hepatic cyst disease, who had undergone cyst resection and inguinal hernia repair at an outside hospital approximately 3 months earlier, presented to the emergency department (ED) with progressively worsening abdominal distention, dyspnea, and bilateral lower extremity edema over several days. He had no primary care physician, took no medications, had no significant alcohol use, and had no known history of cirrhosis or prior paracentesis. 

On presentation, he was afebrile (98.6 °F), tachycardic (heart rate in the 100s), normotensive (109/78 mmHg), and had an oxygen saturation of 95% on room air. Physical examination revealed marked abdominal distension with a positive fluid wave and 3+ bilateral lower-extremity pitting edema. He was not jaundiced. Initial laboratory investigations are summarized in Table 1.

**Table 1:** Laboratory findings on initial presentation. ALT, alanine aminotransferase; AST, aspartate aminotransferase; BUN, blood urea nitrogen; INR, international normalized ratio; TIBC, total iron-binding capacity

Parameter	Patient value	Reference range
Hemoglobin	9.9 g/dL	13.5-17.0 g/dL
Albumin	3.1 g/dL	3.7-5.1 g/dL
Alkaline phosphatase	226 U/L	40-130 U/L
AST	33 U/L	10-30 U/L
ALT	17 U/L	10-62 U/L
Total bilirubin	0.4 mg/dL	0.0-1.2 mg/dL
INR	1.4	0.9-1.2
Creatinine	0.83 mg/dL	0.67-1.23 mg/dL
BUN	12 mg/dL	6-23 mg/dL
Iron	27 µg/dL	50-158 µg/dL
Ferritin	1219 ng/mL	24-370 ng/mL
Transferrin saturation	13%	14%-50%
TIBC	213 µg/dL	240-450 µg/dL

Bedside POCUS identified large pockets of free fluid in the left lower quadrant, inferior to the umbilicus, and in the right lower quadrant. Numerous large, simple-appearing hepatic cysts without clear surrounding free fluid were also seen in the right upper quadrant (Video 1).

**Video 1:** Point-of-care ultrasound of the right upper quadrant at initial presentation. Real-time abdominal sweep demonstrating numerous large, simple-appearing hepatic cysts in the right upper quadrant without clear free fluid between them. The extensive cystic anatomy obscured conventional sonographic landmarks. Free fluid suitable for paracentesis was identified in the lower quadrants.

Computed tomography (CT) of the abdomen and pelvis demonstrated hepatomegaly with innumerable hepatic cysts (up to 10 cm in diameter) and massive ascites (Figure 1). A markedly small-caliber but patent right portal vein was also noted, with normal main and left portal veins. There was no evidence of cirrhosis in the liver tissue between cysts, portal vein thrombosis, or biliary obstruction. The kidneys showed no polycystic changes, with a single simple cyst measuring 1.5 cm in the right kidney (Figure 2).

**Figure 1:**
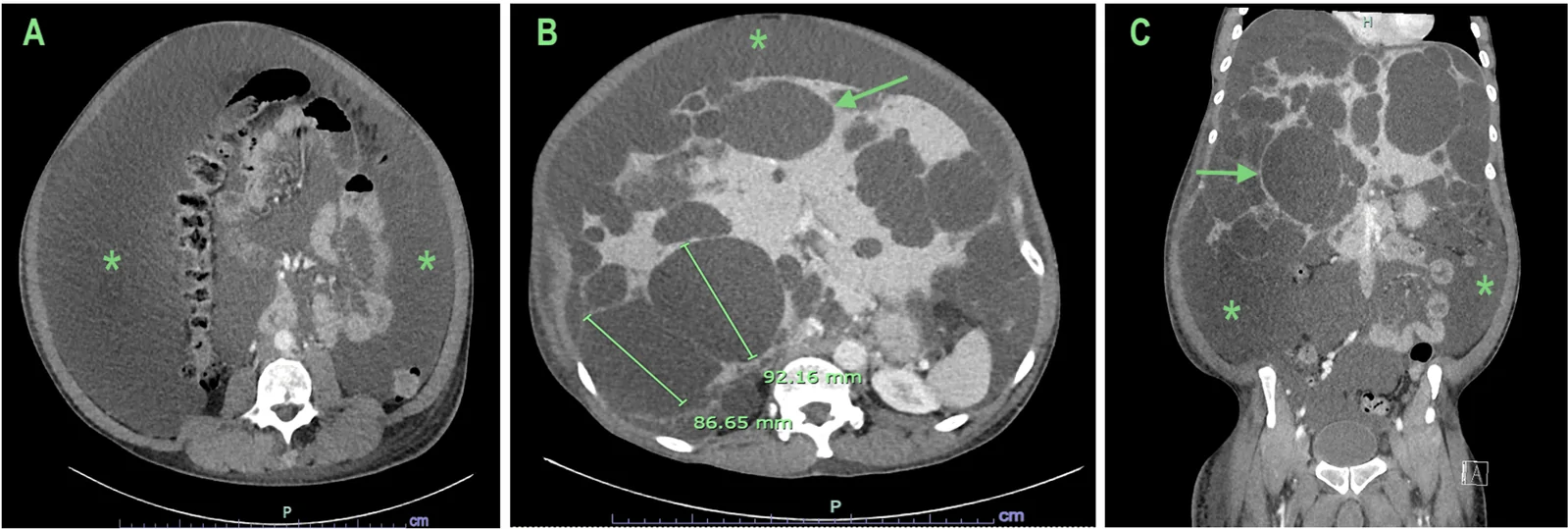
Contrast-enhanced CT of the abdomen demonstrating polycystic liver disease with massive ascites. (A) Axial image demonstrating massive ascites. (B) Axial image showing hepatomegaly with innumerable hepatic cysts (calipers mark representative cysts). (C) Coronal image showing diffuse cystic replacement of an enlarged liver with ascites. Asterisks indicate free intraperitoneal fluid; arrows indicate representative hepatic cysts. No cirrhosis, portal vein thrombosis, or biliary obstruction was identified.

**Figure 2:**
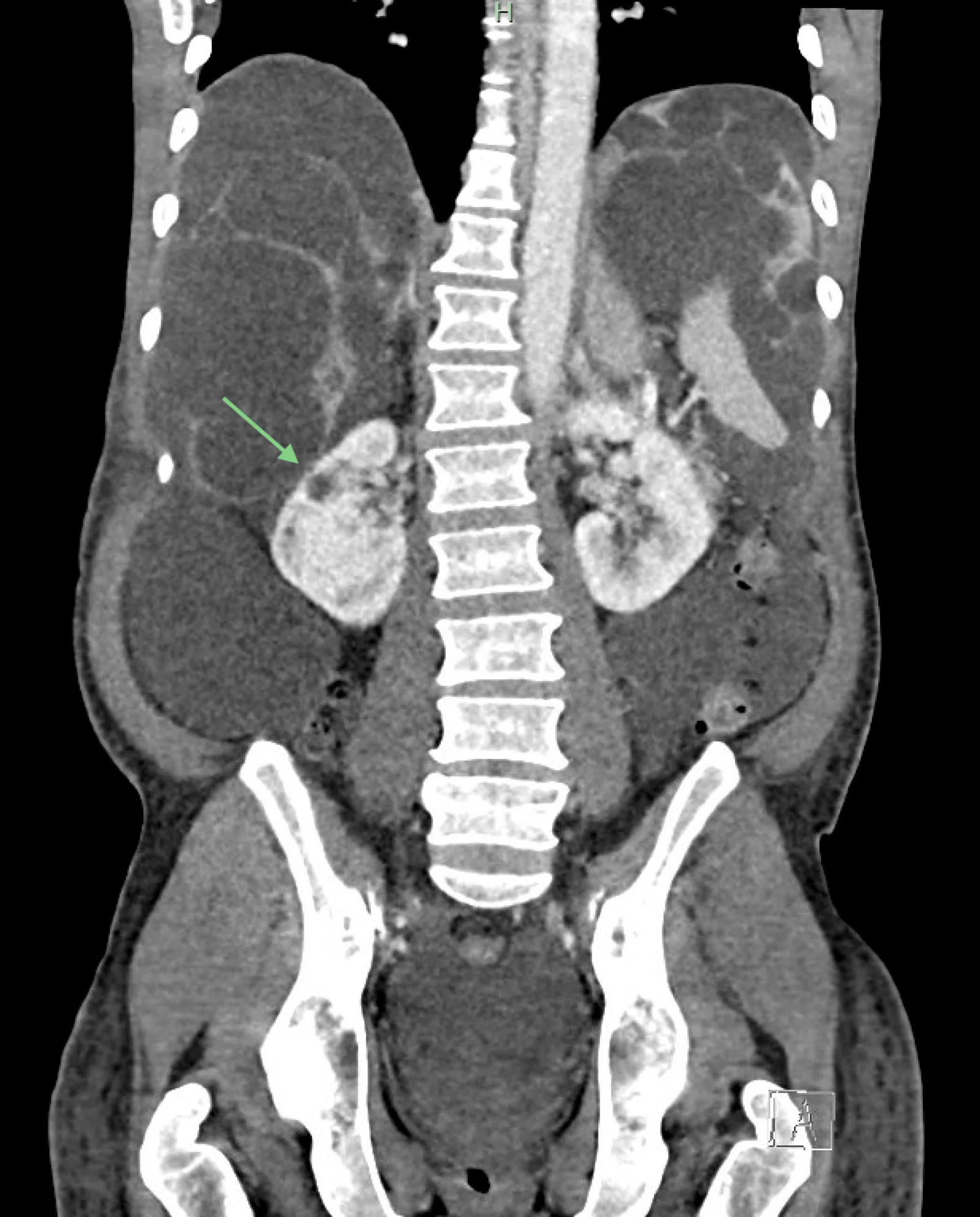
Coronal contrast-enhanced CT showing kidneys without polycystic change. The arrow marks a single 1.5 cm simple cyst in the right kidney.

Paracentesis was performed using POCUS with the static technique to mark the ideal location. The procedure yielded 3.8 liters of clear, straw-colored fluid. Ascitic fluid analysis showed a serum-ascites albumin gradient (SAAG) of 1.1 g/dL, consistent with portal hypertension, and a total protein level of 3.7 g/dL. The ascitic fluid leukocyte count was approximately 400 cells/mm³, with a predominantly monocytic differential. The patient was started on spironolactone 100 mg and furosemide 40 mg daily, with sodium restriction, and was discharged on hospital day 2 with gastroenterology follow-up.

Eighteen days later, he presented to the ED with recurrent tense ascites, dyspnea, and new mild scleral icterus. Repeat paracentesis removed 4.5 liters of fluid. Ascitic fluid analysis showed 395 WBC/mm³, with approximately 16 PMN/mm³ (4% neutrophils), well below the diagnostic threshold for spontaneous bacterial peritonitis (SBP). Gram stain showed no organisms or white blood cells, and cultures were initially negative.

Two days later, ascitic cultures grew viridans group streptococci (broth only), *Streptococcus mitis/oralis* (rare, MALDI-TOF), and *Neisseria flava/perflava/subflava* (broth only). The patient was contacted and asked to return to the hospital, where he was readmitted for further workup of presumed SBP and started on intravenous vancomycin and ceftriaxone.

During the second admission, two additional paracenteses removed a total of 7.7 L of ascitic fluid, and repeat cultures showed no growth at five days. Magnetic resonance imaging (MRI) of the abdomen confirmed innumerable hepatic cysts, some with hemorrhagic or proteinaceous content, and demonstrated diminutive hepatic veins, supporting hepatic venous outflow obstruction in addition to portal vein compression. Alternative causes of ascites were considered but not supported: imaging showed no cirrhosis or portal vein thrombosis, a normal echocardiogram and N-terminal pro-B-type natriuretic peptide (NT-proBNP) excluded cardiac ascites, and negative cytology with no suspicious enhancing lesions on MRI argued against malignancy.

The patient was discharged on oral ciprofloxacin to complete a seven-day course, with continued diuretic therapy and close gastroenterology follow-up. The clinical course and serial ascitic fluid findings are summarized in Table 2.

**Table 2:** Clinical timeline and ascitic fluid findings. ED, emergency department; IV, intravenous; MRI, magnetic resonance imaging; PMN, polymorphonuclear leukocytes; SAAG, serum-ascites albumin gradient; SBP, spontaneous bacterial peritonitis; WBC, white blood cell count

Timepoint	Clinical course	Ascitic fluid findings
~3 months prior	Hepatic cyst resection and inguinal hernia repair (outside hospital)	-
Day 0	ED presentation with abdominal distention, dyspnea, and edema; diuretics started; discharged on hospital day 2	3.8 L removed; SAAG 1.1 g/dL; total protein 3.7 g/dL; WBC ~400/mm³, predominantly monocytic
Day 18	Recurrent tense ascites, dyspnea, mild scleral icterus	4.5 L removed; WBC 395/mm³; PMN 16/mm³ (4%); Gram stain negative
Day 20	Patient called back after positive culture; readmitted for presumed SBP; IV vancomycin and ceftriaxone	Day 18 culture: broth-only growth of viridans group streptococci, *S. mitis/oralis*, and *Neisseria flava/perflava/subflava*
Second admission	Two paracenteses; MRI; discharged on oral ciprofloxacin to complete 7 days	7.7 L removed (total); cultures no growth at 5 days; cytology negative

## Discussion

This case highlights several important clinical considerations in PLD, particularly the pathophysiology of noncirrhotic portal hypertension and the diagnostic limitations of cirrhosis-derived ascitic fluid frameworks. 

Portal hypertension in PLD has been described through three mechanisms: portal vein obstruction, hepatic venous outflow obstruction (HVOO), and inferior vena cava (IVC) syndrome, which may coexist [7,12,13]. The patient's ascitic profile, with a SAAG of 1.1 g/dL and elevated ascitic protein (3.7 g/dL), is more consistent with post-sinusoidal portal hypertension than with cirrhosis, in which ascitic protein is typically low due to impaired hepatic synthetic and endothelial function [12,13]. Although this SAAG lies at the threshold for portal hypertension, imaging provides direct evidence of at least two mechanisms operating simultaneously: a small-caliber right portal vein suggesting cyst compression and diminutive hepatic veins indicating HVOO. Cardiac ascites, the other major cause of high-protein portal hypertensive ascites, was excluded on cardiac evaluation.

The temporal relationship between prior cyst surgery and rapid symptom progression raises the possibility that surgical intervention may have contributed to worsening portal hypertension in an already compromised vascular bed. This is consistent with the well-described risk of postoperative ascites following fenestration in diffuse PLD, which may reflect further disruption of venous or lymphatic drainage [10,11].

A central issue in this case was the interpretation of ascitic fluid cultures. Despite a markedly low PMN count (16/mm³) and negative Gram stain, the patient was treated for presumed SBP following broth-only growth of low-virulence commensal organisms on initial culture. Broth-only isolates are frequently contaminants. In a prospective analysis of 356 such cultures, up to 73% were found to be nonpathogenic, and among streptococci specifically, only 42% were clinically significant [14]. In this case, repeat paracenteses demonstrated no growth, and the initially identified organisms were consistent with oropharyngeal flora. These findings strongly suggest specimen contamination or transient bacterascites rather than true peritoneal infection.

The initial presumption of SBP led to treatment escalation with broad-spectrum intravenous antibiotics, likely reflecting the reflexive application of a cirrhosis-based diagnostic framework to a noncirrhotic disease process. Current SBP criteria were validated in low-protein, opsonin-poor environments, mirroring typical cirrhotic ascites [8,9]. In contrast, this patient's elevated ascitic protein concentration suggests preserved opsonic activity and a fundamentally different infection risk profile [15,16]. In the context of this presentation, a diagnosis of bacterascites (defined as a positive culture with a PMN count below 250/mm³) could have been considered [17]. Even so, current guidelines suggest that patients with bacterascites in the absence of signs of infection should not receive antibiotics, as most cases self-resolve or represent contamination [8,17]. In this case, the absence of neutrocytic ascites argues against SBP, and the broth-only growth pattern with negative repeat cultures argues against true bacterascites, making specimen contamination the most likely explanation. This case underscores the need for more nuanced diagnostic paradigms for noncirrhotic ascites.

Finally, this case underscores the utility of POCUS in patients with advanced PLD. POCUS allowed rapid identification of diffuse hepatic cystic disease and large-volume ascites. In addition, POCUS provided guidance for safe paracentesis in the setting of an enlarged cystic liver that obscured traditional anatomical landmarks. Given that ultrasound can detect as little as 100 mL of ascitic fluid (compared with significantly lower sensitivity on physical examination), it represents a critical tool in both diagnosis and procedural management [18].

Limitations of this case include the absence of genetic testing to confirm ADPLD, although the lack of renal cystic disease on imaging (Figure 2) favors isolated ADPLD over ADPKD. External operative records were also unavailable to fully characterize prior surgical intervention. Further, liver biopsy was not performed, and thus congenital hepatic fibrosis, an independent etiology of portal hypertension, cannot be excluded.

## Conclusions

This case illustrates the clinical complexity of severe polycystic liver disease presenting with recurrent massive noncirrhotic ascites. It highlights the multimechanistic nature of portal hypertension as a driver of ascites and the potential contribution of prior surgery to hemodynamic compromise. The case further demonstrates how reflexive application of cirrhosis-derived diagnostic frameworks, particularly SBP criteria, to noncirrhotic ascites can lead to diagnostic misclassification and unnecessary treatment escalation. As PLD-associated ascites becomes increasingly recognized, greater attention should be given to interpreting ascitic fluid results within the appropriate pathophysiological context. Further research is needed to establish evidence-based diagnostic and management frameworks specifically tailored to noncirrhotic ascites. Finally, this case reinforces the utility of POCUS as both a diagnostic and procedural adjunct in patients whose hepatic anatomy is altered by extensive cystic disease.
